# Movement patterns of a small-bodied minnow suggest nomadism in a fragmented, desert river

**DOI:** 10.1186/s40462-024-00490-w

**Published:** 2024-07-31

**Authors:** Martinique J. Chavez, Phaedra Budy, Casey A. Pennock, Thomas P. Archdeacon, Peter D. MacKinnon

**Affiliations:** 1 Department of Watershed Sciences, Utah State University, Logan, UT USA; 2 American Southwest Ichthyological Researchers, Albuquerque, NM USA; 3 U.S. Geological Survey, Utah Cooperative Fish and Wildlife Research Unit, Utah State University, Logan, UT USA; 4 Department of Evolution, Ecology, and Organismal Biology and Aquatic Ecology Laboratory, The Ohio State University, Columbus, OH USA; 5 U.S. Fish and Wildlife Service, New Mexico Fish and Wildlife Conservation Office, Albuquerque, NM USA

**Keywords:** Pelagic-broadcast spawning, Nomadic movement, Passive Integrated Transponder (PIT) tags, Small-bodied minnow, Fragmentation

## Abstract

**Background:**

Unfettered movement among habitats is crucial for fish to access patchily distributed resources and complete their life cycle, but many riverscapes in the American Southwest are fragmented by dams and dewatering. The endangered Rio Grande silvery minnow (*Hybognathus amarus,* RGSM) persists in a fragmented remnant of its former range (ca. 5%), and its movement ecology is understudied.

**Methods:**

We tracked movements of hatchery-reared RGSM, tagged with passive integrated transponder tags, using stationary and mobile antennas from 2019 to 2022. We quantified probability of movement and total distance moved by RGSM released above and below a dam. We then assessed how well two prevailing riverine movement theories (i.e., restricted movement paradigm [RMP] and colonization cycle hypothesis [CCH]) explained RGSM movement patterns.

**Results:**

We detected 36.8% of released RGSM (*n* = 37,215) making at least one movement. Movements were leptokurtic and substantially greater than expected based on the RMP for both stationary (1.7–5.9 m) and mobile (30.3–77.8 m) individuals. On average, RGSM were detected at large for 75 days and moved a total of 12.2 rkm within a year. The maximum total distance moved by RGSM was 103 rkm. Similarly, we observed a multimodal distribution of detected range sizes with a mean detected range of 2.4 rkm and a maximum detected range of 78.2 rkm. We found little support for an upstream movement bias, as expected under the CCH, and most movements (74%) were directed downstream.

**Conclusions:**

Our data suggest RGSM are highly mobile, with the ability to make long-distance movements. Neither movement theory adequately described movement patterns of RGSM; instead, our findings support a nomadic movement pattern and an apparent drift paradox matching recent studies of other pelagic-broadcast spawning minnows where populations persist upstream despite experiencing downstream drift as larvae. Resolution of the drift paradox may be achieved through further, targeted studies into different aspects of the species’ life history. Quantification of RGSM movement provides crucial insights into the species’ movement ecology and may help define the appropriate scale of recovery efforts.

**Supplementary Information:**

The online version contains supplementary material available at 10.1186/s40462-024-00490-w.

## Background

Many riverscapes are fragmented by dams and water diversions that modify natural flow regimes, degrade habitats, and contribute to the imperilment of native freshwater fishes. Dam construction and water extraction have compromised habitat quality, riverine connectivity, and restricted fish movement [1], contributing to widespread range reductions and local population declines [2, 3]. Fragmentation exacerbates the effects of environmental disturbance, such as drought, and in extreme cases can lead to recruitment failure, prevention of recolonization from downstream populations, and ultimately extirpation [4, 5]. Conserving mobile species is often difficult [6], but quantifying movement patterns can provide important information to guide management actions, such as the need to restore longitudinal and lateral connectivity among habitats during critical life-stages or guiding design of fish passage at in-stream barriers [7]. There is limited quantitative information on movement ecology for many species, despite its potential to inform management of riverine fishes [8]. A recent study classifying the migratory tendency of 1,250 North American fishes suggested 44% lack sufficient movement data [9]. Quantifying movement patterns of more species and framing results within broader movement theories will contribute to a better understanding of riverine fish ecology and help guide appropriate restoration efforts at ecologically relevant scales [10].

For over three decades, the prevailing thought of riverine fish movement ecology was that fish generally displayed restricted movement, whereby individuals remain in relatively small home ranges for the entirety of their lifespan [11]. Gowan et al. [12] formalized this idea as the restricted movement paradigm (RMP), but Rodríguez [13] noted that most studies of fish movement searched for marked fish mostly within or very near habitats where fish were released, underestimating the mobility of stream fishes. Now, movement within fish populations is thought to be more heterogeneous with most individuals having a ‘stationary’ tendency and a smaller component of ‘mobile’ individuals [13–15]. In a metanalysis of the movement of 40 riverine fishes, Radinger and Wolter [15] presented a model of the RMP including heterogenous movement tendencies to predict the expected movement of stationary and mobile components of populations based on a suite of characteristics. This model has been validated for a few small-bodied species (e.g., [16, 17]); however, as movement studies are being conducted on a broader range of species, more tests are required to assess the generality of the RMP (e.g., [18]).

Another theoretical framework used to explain patterns of fish movement is the colonization cycle hypothesis (CCH). The CCH assumes early life stages of riverine organisms experience some degree of downstream drift and older individuals must make net upstream movements to offset this displacement and maintain population persistence [19, 20]. Thus, some adults must undertake upstream movements to prevent local extirpation from upstream habitats. Based on early work by Moore [21], freshwater minnows in the pelagic-broadcast spawning (PBS) guild have been assumed to follow the CCH. Pelagic-broadcast spawning is a reproductive strategy somewhat unique for riverine freshwater fishes [22], and likely promoted survival of offspring in the historically dynamic and harsh conditions of large, arid riverscapes [22, 23]. Species in the PBS guild release nearly neutrally-buoyant, non-adhesive eggs into the water column that drift passively into a variety of downstream habitats [21, 24]. Drifting is likely an efficient strategy for maximizing the chance that propagules find appropriate nursery habitats, particularly in heterogeneous and rapidly changing environments [22]. There is some evidence for upstream movement by PBS fishes (e.g., [18, 25, 26]), but evidence of synchronized mass upstream movement to counteract downstream drift is limited to anecdotal observations [27, 28]. Movement information for small-bodied fishes in larger rivers is lacking generally due to limitations of marking and recapturing small fishes in larger rivers [29] More research is needed, across spatiotemporal scales relevant to management efforts (e.g., [23]), to understand the importance of upstream movements for the long-term persistence of PBS fishes in fragmented rivers.

Species of PBS fish were formally widespread and numerically dominant in rivers across the Great Plains and the Rio Grande Basin in the Southwestern USA [30]. The Rio Grande in New Mexico, USA, historically supported five PBS fishes, and all except the Rio Grande silvery minnow (*Hybognathus amarus,* RGSM) are extirpated. Fluctuations in the occurrence and density of RGSM over the past three decades are closely related to variation in spring and summer flows [31, 32], and nearly annual stockings of hatchery-reared fish are required to maintain populations in the wild. Extensive geomorphic and hydrologic modifications along the Middle Rio Grande have compromised habitat quality, complexity, and longitudinal and lateral connectivity [33]. Bank modifications for flood control have increased river incision and further reduced habitat complexity [34, 35], contributing to diminished refuge areas during periods of drought [36]. The active channel width of the Rio Grande has been decreasing since the 1930s, and the river has almost completely lost connection to the historical floodplain [35], coinciding with a decrease in flood frequency, amplitude, and total water volume in the river [37]. Frequent river drying, due to seasonal drought and water withdrawals to meet agricultural and municipal demands, contributes adversely to recruitment and population persistence [31, 38]. Consequently, there is a low effective population size of wild RGSM in the Middle Rio Grande [39, 40], and the negative impacts to demographic resilience and genetic diversity are managed through the hatchery program [41, 42].

Since being listed as federally endangered, RGSM is the subject of numerous recovery efforts which include: (1) augmenting wild populations with hatchery-reared fish, (2) attempting to transport stranded RGSM upstream during periods of river drying, and (3) restoring flood-plain connectivity in the Middle Rio Grande [43]. In addition to these recovery actions, long-term demographic and genetic population monitoring occurs across the species’ remaining range [40, 42]. However, long-term recovery of this species will not be achieved without addressing and ameliorating the adverse effects of the extensive anthropogenic modifications to the river, including extensive river drying and fragmentation [31, 33]. The fragmentation-induced declines of other broadcast spawning minnows [4] suggests upstream dispersal may be important for long-term population persistence; however, data on RGSM movement patterns are limited.

In this study, we capitalized on the ability to use large numbers of tagged, hatchery-reared RGSM coupled with re-detection efforts across broad spatiotemporal scales (days-years; 10^2^–10^5^ m; [23]) to explore and better understand RGSM movement patterns. Our goal was to quantify movement of RGSM and identify relationships between movement patterns and biological and environmental variables (i.e., river discharge, season, body length). Additionally, we assessed how well RGSM movement patterns matched with patterns predicted by prevailing movement theories (RMP, CCH). We hypothesized that RGSM movement distances would be greater than expected under the RMP because of previously observed long-distance movements [44]. If the CCH explains RGSM movement patterns, movement should be biased in an upstream direction to counteract the downstream drift of propagules. Given the unique reproductive mode of RGSM, we further hypothesized there would be a strong positive relationship between movement distances and increased spring flows that preceded spawning.

## Methods

### Study area

**Fig. 1:**
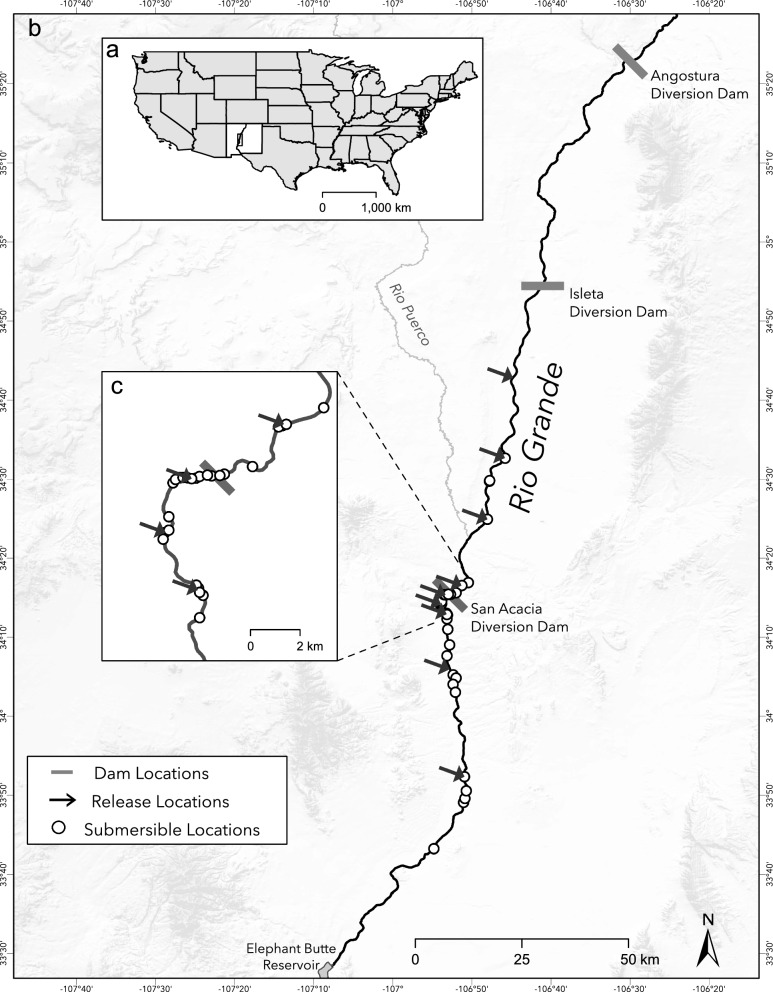
Overview of study area located in New Mexico (panel **a**), showing locations of fish releases and locations of submersible antennas (panel **b**), and the area surrounding the San Acacia Diversion Dam (panel **c**)

The Rio Grande originates in the San Juan Mountains of southern Colorado, draining over 550,000 km^2^ in the United States and Mexico [45]. The fourth longest river in the United States, the Rio Grande runs ~ 3040 river kilometers (rkm) from southern Colorado, southward through New Mexico, forming the international border between Texas and Mexico. We conducted our study along two reaches of the Rio Grande located within the Rio Grande Valley in central New Mexico (Fig. 1). The Middle Rio Grande (MRG hereafter) flows about 330 rkm between the upstream impoundment of Cochiti Dam and downstream into Elephant Butte Reservoir [46]. Historically, the MRG was a wide, braided, aggrading system with sand substrate and expansive floodplains and wetlands during periods of high flow [47]. The contemporary MRG has a highly modified flow regime and > 80 rkm dries annually, with current total annual water volume of northern reaches estimated to be 95% lower than historical volume [37]. Diversion dams along the MRG divert water into a complex system of ditches, drains, and conveyance canals, facilitating extensive irrigation throughout the MRG valley.

These diversion dams delineate the MRG into four reaches (*upstream to downstream*): Cochiti (36.2 rkm), Angostura (65.6 rkm), Isleta (85.5 rkm), and San Acacia (102.3 rkm). Our study area was limited to the three lowermost reaches, spanning about 250 rkm from Angostura Dam to Elephant Butte Reservoir. The length of the San Acacia reach varies temporally based on the water level of the reservoir. The MRG in the Angostura, Isleta, and San Acacia reaches is primarily shallow with sand substrates. An inflatable-bladder dam (Alameda Diversion) within the Angostura reach has a fish bypass structure. Long-term population monitoring occurs across these three reaches within the MRG, and the highest abundance of RGSM is often within the San Acacia reach [43, 48]. A large portion (~ 90%) of the San Acacia reach is designated as critical habitat for RGSM [43].

### Fish tagging and release

The U.S. Fish and Wildlife Service’s Southwestern Native Aquatic Resources Recovery Center in Dexter, New Mexico and Albuquerque BioPark Aquatic Conservation Facility provided hatchery-reared RGSM used in this study. Fish from the Aquatic Conservation Facility were age-5 brood-stock originally collected as eggs and tagged at the New Mexico Interstate Stream Commission Los Lunas Silvery Minnow Refugium (*n* = 1,701). Fish provided by the U.S. Fish & Wildlife Service were age-1 captively-spawned fish and tagged at the Southwestern Native Aquatic Resources and Recovery Center (*n* = 36,649). We surgically implanted fish with a Passive Integrated Transponder (PIT) tag (Biomark Model APT12; 134.2 kHz, 12.50 × 2.07 mm, 0.102 g) following methods outlined in Archdeacon et al. [49]. We tagged hatchery-reared RGSM > 44 mm standard length (SL). In addition to PIT tag identification codes, we recorded SL of each fish during tagging. Then, we placed tagged RGSM into recovery tanks immediately after tag implantation and held them for a minimum of two weeks prior to release to remove fish that died or lost their tag. Most fish were in good condition after tagging, and a 5–10% tag loss was recorded in the two-week holding period prior to release. We only released fish that appeared to be in good condition and retained a PIT tag. Between 2019 and 2022, we released eight batches of PIT-tagged RGSM (Table 1). Release locations were selected based on truck access as well as proximity to diversion dams (Table S1).

**Table 1:** Rio Grande silvery minnow released between 2019 and 2022 in the Middle Rio Grande of New Mexico, USA showing the year and month released, number of submersible antennas deployed, release location (river kilometer, rkm), and the number of PIT-tagged individuals released

Year	Month	Submersibles deployed	Release location (rkm)	Number released
2019	Mar	20	180.9, 182.9, 186.0, 191.5	1701
2019	Nov	20	180.9, 182.9, 186.0, 191.5	9875
2020	Mar	20	180.9, 182.9, 186.0, 191.5	7916
2021	Mar	11	139.4, 166.2, 183.0, 191.5, 211.1	4865
2021	Dec	11	183.1, 191.5, 227.4, 250.9	9131
2022	Mar	11	191.5, 227.4, 248.6	2991
Total				37,215

### Fish detection

We used semi-permanent submersible, floating raft-mounted, and mobile wand antennas to detect tagged RGSM. We installed multiple semi-permanent submersible PIT tag antennas (Biomark; Boise, Idaho, USA) in the river channel that detect tags as fish swim or drift over an antenna. Submersible antennas had a read range of 31–75 cm (0.91 diameter). We maintained antennas in the river throughout the study period. Occasionally, we removed and relocated antennas due to placement and maintenance logistics (e.g., changes in flow magnitude). In such instances, antennas were relocated within < 1 rkm of their initial deployment location in effort to maintain consistency across years. There were 7–20 antennas deployed at any given time (Table 1).

We actively scanned for tagged RGSM using floating Passive Integrated Transponder Portable Antenna Systems (PITPASS; [50]). The PITPASS system is a raft mounted system and includes an integrated Global Positioning System (GPS), and a data recorder (Fig. S-1). Each PITPASS system consists of a 6 m × 1.2 m antenna with a vertical read range of up to 101.0 cm which is ideally suited for shallow river channels like that of the MRG. We simultaneously launched three PITPASS equipped rafts (each with a 6 m × 1.2 m antenna) at the same location, making multiple passes during each sampling trip (*n* = 2–5 passes of each segment per float). We considered active detection within a river section, typically in one day, to be one pass. Each raft covered a distinct section of the river channel: river right, center channel, or river left and drifted simultaneously along their designated position in the river channel with rafts oriented perpendicular to the flow of the river. The duration of trips (2–12 days) and river extent sampled varied based on flow and trailer access. We conducted multiple active detection trips in 2019, 2021, and 2022, including initial trips within 5 days following fish releases (Table S-2). Our efforts in 2020 were limited to one active detection trip because of the COVID-19 pandemic. Given summer low flows and intermittency in the MRG, shallow, low-velocity habitats (e.g., backwaters) were not always accessible by raft. When these habitat types were encountered on float trips, we used portable PIT tag antennas (wands), to actively detect RGSM. Detections made by floating raft-mounted antennas and portable wand antennas were considered active detections. Portable wand antennas had a read range of 41.0–56.0 cm and were taken on all float trips each year.

### Movement metrics and potential correlates

To assess movement patterns in relation to operation of irrigation withdrawals, we categorized two seasons based on the operational months of the MRG irrigation system (irrigation or off). The irrigation system is operational from March to October and off from November to February. The early irrigation season coincides with peak spring runoff from March to June, and the late irrigation season coincides with increased irrigation demands and subsequent river drying from July to October. We calculated movement metrics for individuals with at least one movement (i.e., two detections after release). To account for the typical life span of hatchery-reared RGSM (generally < 12 months after release; [32]) and reduce the effects of ‘ghost tags’ (e.g., [50]), we limited analyses to detections that occurred within 365 days of a fish being released. We used ArcGIS Pro to snap detection locations within the nearest 0.1 rkm to the sampled river network to allow calculation of movement distances between detections and facilitate calculation of movement metrics (Fig. 1). To describe RGSM movements, we calculated several metrics including: total distance moved, net distance moved, and detected range size. To account for the tendency of hatchery-reared RGSM to immediately move downstream following release [42, 44], we also calculated metrics excluding detections within the first week after release (treating the first detection at least one week after release as the starting point). When the first week of detections following release were excluded from analyses, patterns were largely unchanged. As such, we treated the first detection after release as the starting point when calculating movement metrics. We calculated total distance moved as the sum of distances between all successive detection locations regardless of direction (upstream or downstream). We calculated net distance moved as the net displacement between the first detection location after release and last detection location in either the upstream or downstream direction for each individual. We calculated total and net distance moved both across seasons and for each season separately. Finally, for detected range size, we calculated the distance between the most downstream and most upstream detection, [51] which represents the total linear extent of the riverscape used over the course of 1 year [51]. We calculated cumulative days at large as the number of days between the first and last detection for each individual within a season. We calculated the mean daily discharge for the corresponding days an individual was at large within each season using U.S. Geological Survey (USGS) gage data from San Acacia (USGS 08354900; USGS 2023). All data manipulation and analyses were performed using R version 4.1.2 [52].

We tested the kurtosis of the distribution of total distances moved using an Anscombe-Glynn’s test [53]. We related spatiotemporal variation in movement patterns to biological and environmental covariates using mixed effects models. We calculated the total distances moved by season, which included zeros for individuals detected multiple (> 2) times at the same location within a season (i.e., detected but not moving) and positive values for all other individuals within a season (i.e., detected and moving). Because seasonal movement data were positive-only, continuous, and contained many zeros, we first modeled the probability of a fish moving (1) or not (0) using a generalized linear mixed effects model with a binomial distribution (link = logit). Then, we modeled the log-transformed, non-zero total distances moved using a linear mixed effects model. In both models, we included year detected (categorical), cumulative days at large (continuous), mean daily flow (continuous), body length at release (standard length, SL mm; continuous), and the additive and interactive effects of irrigation season (categorical) and release location (above or below San Acacia Diversion Dam; categorical) as fixed effects in the model. We included individual PIT tag ID as a random effect to account for multiple observations of individual fish. We built mixed effects models using the R package ‘lme4’. We screened all covariates for multicollinearity and found minimal correlations between covariates included in the models (all variance inflation factor < 5). We calculated marginal *R*^2^ and conditional *R*^2^ for mixed effects models, which represent the variance explained by fixed effects alone as well as the variation explained by both fixed and random effects (i.e., variation among individual fish), respectively [54]. Finally, we ensured model assumptions were reasonably met by inspecting residual plots.

### Restricted movement paradigm

We used the total distance moved metric in the R package ‘fishmove’ to test the hypothesis that RGSM would move farther distances than expected under the RMP [15, 52]. This function predicts the average distance moved for the stationary and mobile component of a population using a multiple regression with body length, caudal fin aspect ratio (A = height^2^/surface area), stream order, and time at large as predictor variables. We parameterized the expected movement model for individuals across years (2019–2022) using median body length (63.0 total length, TL mm; estimated from SL mm using a formula developed by Horwitz et al. [55]) of tagged individuals, the stream order of the Middle Rio Grande (7th order), caudal fin aspect ratio reported in the literature [56] and the median number of days between release date and date of last detection for individuals in our study (92 days). We fit a double normal distribution to our observed total distances moved by individual RGSM to estimate the distances moved by the stationary and mobile components. We then assessed whether our observed movement distances fell within the 95% confidence intervals of the expected movements from the RMP model.

### Colonization cycle hypothesis

To test if RGSM exhibited upstream movement bias associated with spawning as expected by the CCH, we used a two-way analysis of variance (ANOVA) correcting for unbalanced numbers of detections by year using a sum of squares type III model [57]. We used net distance moved as the response variable and included the additive effects of season and year and their interactive effect. We also calculated effect sizes (*η*^2^) for each term in the model using the R package ‘effectsize’. We again used residual plots to ensure model assumptions were reasonably met.

## Results

We detected RGSM across a large portion (70%) of the MRG, spanning 176 rkm of river fragmented by two diversion dams. We collected a total of 96,152 detections throughout our study period. Most detections were made by submersible antennas (*n* = 83,342), which were operated nearly continuously throughout the study, followed by floating antennas (*n* = 12,771) and mobile wand antennas (*n* = 39). Of the 37,215 PIT-tagged RGSM released between 2019 and 2022, 13,706 unique PIT tags were detected at least twice after release, a 36% resight rate. Forty-eight percent of detections occurred within the first two months following a release. Fish were at large in the study system for an average of 25 days (with a range of 15–365 days). Throughout the study period, 57% of all detections occurred within 15 rkm downstream of the San Acacia Diversion Dam, where most (54%) fish were released.

### Movement metrics and potential correlates

**Fig. 2:**
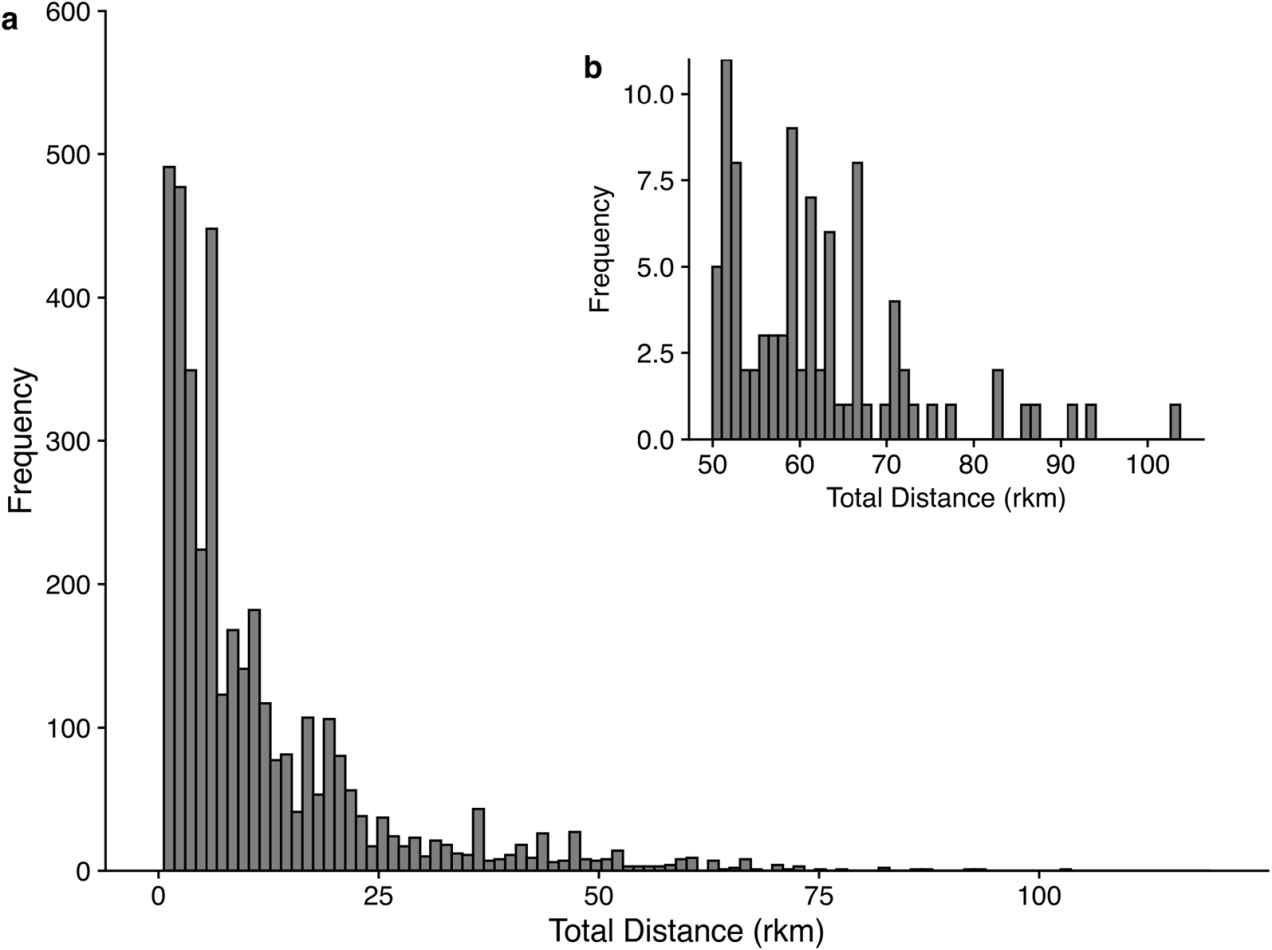
Total distance moved (absolute value, rkm), both upstream and downstream, by PIT-tagged Rio Grande silvery minnow in the Middle Rio Grande from 2019 to 2022 with total distance on the x-axis and frequency of the y-axis (panel **a**) and panel **b** showing the movement rate with total distance moved (absolute value, rkm) on the x-axis and the cumulative number of days since release on the y-axis

Rio Grande Silvery Minnow movement was leptokurtic, and fish moved long distances both upstream and downstream regardless of irrigation season (Fig. 2). The distribution of total distances moved was significantly leptokurtic and highly skewed (kurtosis = 10.4, *z* = 26.5, *P* < 0.001). A total of 91 individuals moved over 50 rkm within one year of release, and 30% of individuals in the study moved farther than the mean total distance of 10.4 rkm. The maximum total distance we observed a fish moving was 103.0 rkm (Fig. 2). The mean total distance moved was 19.8 rkm for individuals moving upstream and 15.2 rkm for individuals moving downstream. The mean total distance moved was 1.8 rkm during the off season and 1.5 rkm during irrigation season (Fig. S-2). The highest maximum total distance moved occurred in the off season; however, the distribution of total distances moved overlapped substantially between both the irrigation and off seasons.

**Fig. 3:**
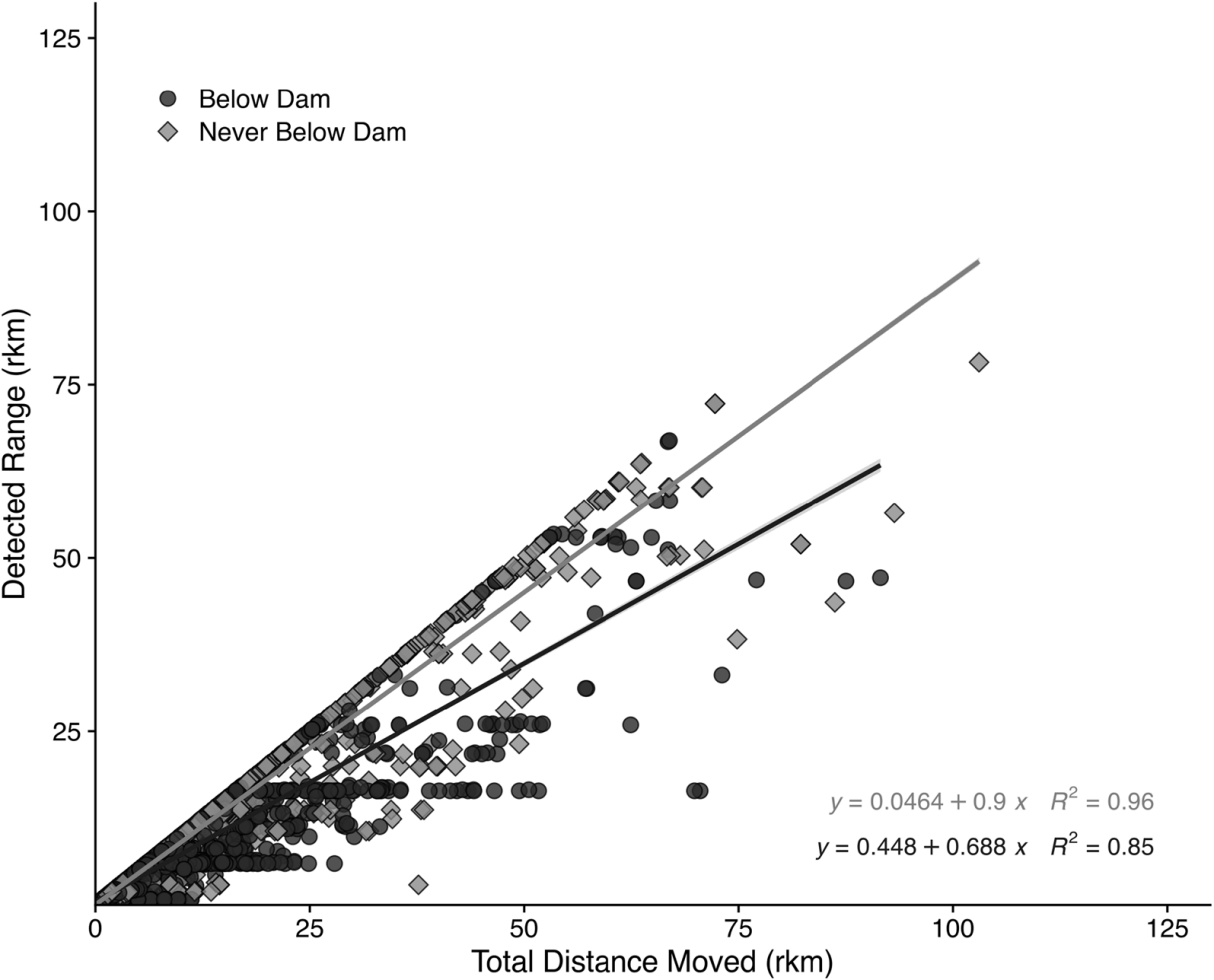
Total distance moved (rkm) by detected range size (rkm) of Rio Grande silvery minnow between 2019 and 2022 in the middle Rio Grande, with shades of grey representing release location in relation to San Acacia Diversion Dam. The individual with the largest detected range of 78.2 rkm moved a total distance of 103 rkm. The linear regressions lines and equations represent the corresponding slope of each group, released above or below the dam by shades of grey

Individuals in our study had larger total distances moved than their detected ranges, suggesting RGSM are moving at much finer scales within their detected range. The mean detected range size of individuals in our study was 2.4 rkm, and the maximum was 78.2 rkm (Fig. 3). Tagged RGSM used 21% of the study area, and individuals with the largest detected ranges (*n* = 1,176) used 44% of the study area. Over half of individuals (52%) had detected ranges within 1.0 rkm of their total distance moved.

**Fig. 4:**
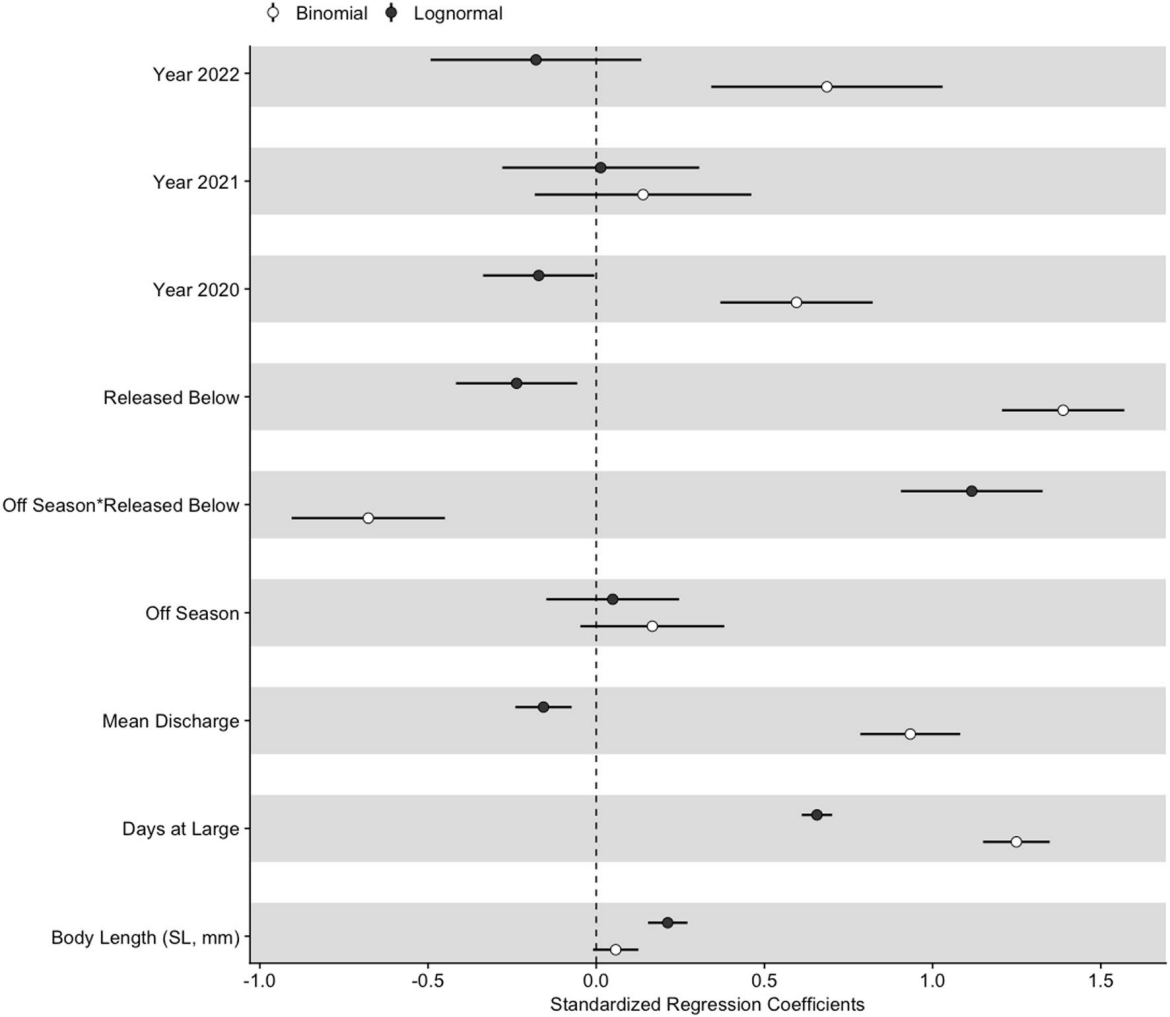
Covariates included in a binomial logistic regression and zero-truncated lognormal model assessing variation in Rio Grande silvery minnow movement patterns by season on the y-axis and standardized regression coefficients on the x-axis, with shade representing model and horizontal bars showing the 95% confidence interval. The number of cumulative days at large and the interaction between season and release location (above or below San Acacia Diversion Dam) were significant effects (*P* < 0.001) in both models

**Fig. 5:**
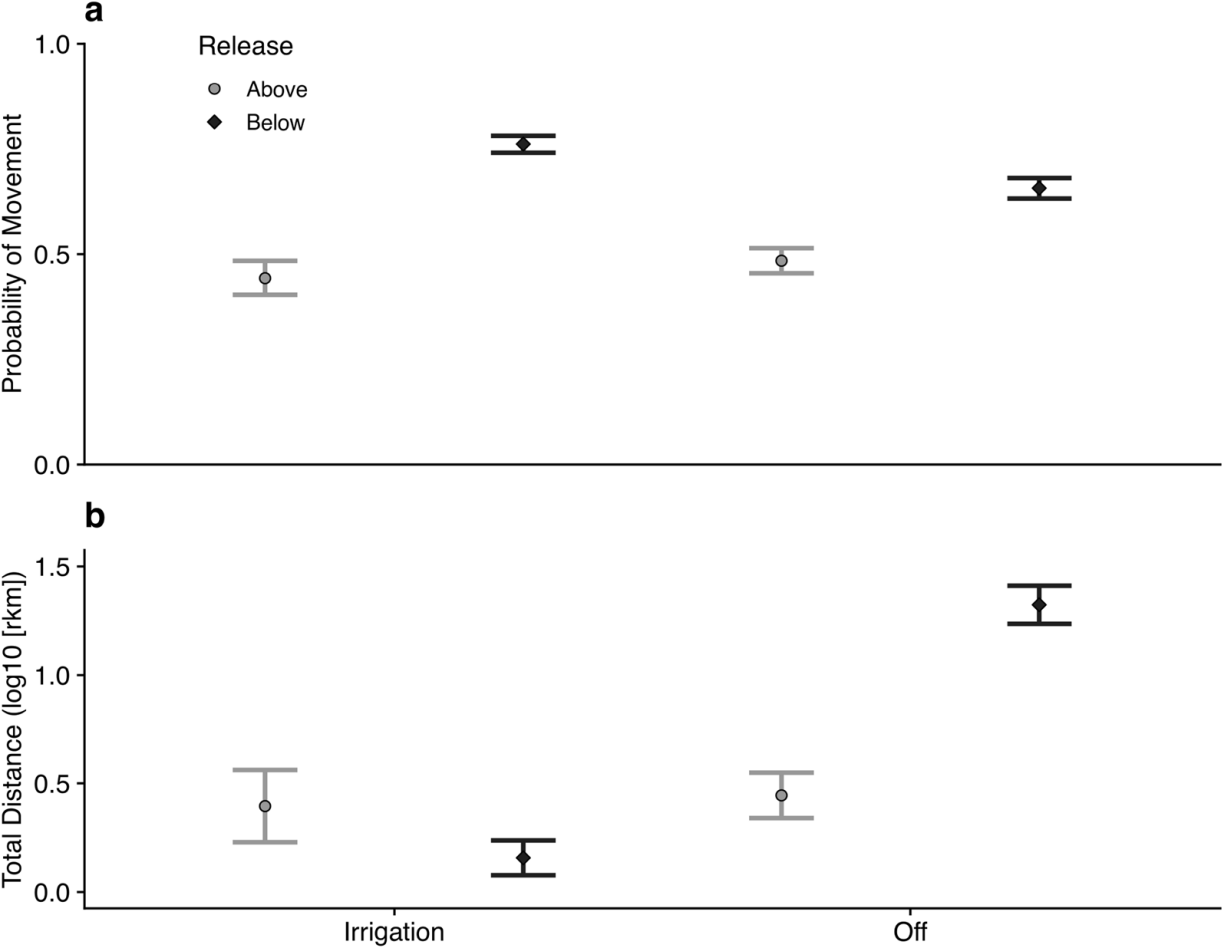
Significant interaction between season and release location (above or below San Acacia Diversion Dam) produced by binomial logistic regression (panel **a**) with probability of movement (0 = no movement; 1 = movement) on the y-axis and season on the x-axis and significant interaction produced by zero-truncated lognormal model (panel **b**) assessing the positive total distances moved by season, with log-transformed total distances on the y-axis and season on the x-axis. Shaded shapes correspond to release location and error bars represent 95% confidence interval

Mixed effects models revealed spatial and temporal variation in the probability of moving and total distance moved. The probability of moving and total distance moved varied among years, with flow, days at large, and seasonally based on release location (Fig. 4). The generalized linear mixed effects model with a binomial distribution revealed fish were more likely to move during periods of higher flow and with more time at large, with marginal *R*^2^ = 0.45 (Table S-3). The interactive effect of release location and irrigation season was statistically significant, whereby fish released below San Acacia Diversion Dam were more likely to move than fish released above in both seasons, and the magnitude difference was greater during irrigation season (Fig. 5). When considering only the fish that moved, coefficients of predictors of total distance moved varied somewhat in sign from coefficients from the logistic regression (Fig. 4). Results of the linear mixed effects model suggested total distance moved was higher with more time at large but was lower with higher flow, with an marginal *R*^2^ = 0.19 and a conditional *R*^2^ = 0.20 (Table S-4). The interaction between irrigation season and release location was statistically significant, whereby fish released above the dam moved similar distances in both seasons, and fish released below the dam moved substantially shorter distances during irrigation season than during the off season (Fig. 5).

### Restricted movement paradigm

Individuals in our study moved orders of magnitude greater than expected under the RMP. The fitted mean expected movement distances of the stationary and mobile component of the population were < 0.01 rkm and 0.05 rkm, respectively. The double normal distribution fitted to the observed data across all years produced movement distance estimates of 0.24 rkm and 23.94 rkm for the stationary and mobile components, respectively.

### Colonization cycle hypothesis

**Fig. 6:**
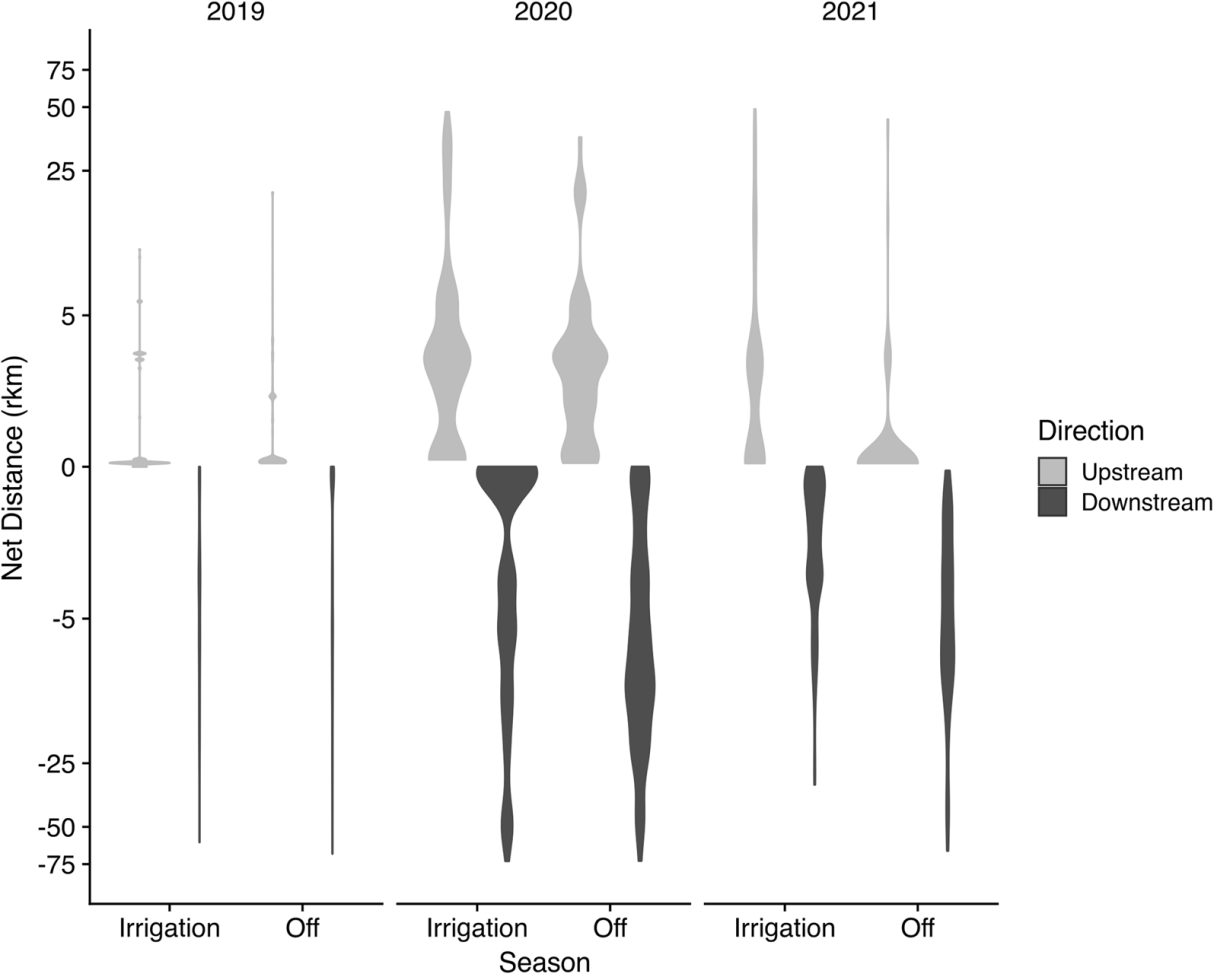
Violin plots of net distance moved downstream or upstream (rkm, pseudo-log scale) by PIT-tagged Rio Grande silvery minnow by year and season (Irrigation = March–October; Off = November–February). The width of each violin plot corresponds with data density and frequency of net directional movements. Net distances moved were generally farther in the downstream direction across years and seasons

Movement patterns of RGSM were inconsistent with those expected under by the CCH. We found no evidence of upstream bias in RGSM movement (Fig. 6). The maximum distance moved downstream was 72.5 rkm, and the maximum distance moved upstream was 48.8 rkm. Tag movements were generally directed downstream, with 79% of all detected movements directed downstream (Table 2). There was a statistically significant interaction between season and year (Type III ANOVA, *F*_12,137_ = 34.94, *df* = 3, *P* < 0.001); although, our power to detect differences was large (*n* = 12,144) and effect sizes for all terms in the model were small (all *η*^*2*^ < 0.05; Table 3). Regardless, net distances moved were generally farther in the downstream direction, and the frequency of downstream movements were consistently larger across years and seasons; the opposite pattern predicted by the CCH.

**Table 2:** Summary statistics of annual detections and net movement distances of Rio Grande silvery minnow in the Middle Rio Grande of New Mexico, USA between 2019 and 2022 by direction

Year	Direction	Count	Mean	SD	Median
2019	Downstream	4963	4.81	7.12	2.89
	Upstream	816	1.48	3.38	0.16
2020	Downstream	3773	8.74	13.90	2.90
	Upstream	1070	5.60	8.59	3.06
2021	Downstream	605	4.53	7.05	2.89
	Upstream	384	2.81	7.18	0.10
2022	Downstream	305	7.84	10.70	5.07
	Upstream	236	1.38	3.83	0.10

**Table 3:** Results of two-way ANOVA using a sum of squares type III model for net distance moved upstream and downstream by Rio Grande silvery minnow by year and season (*n* = 12,144)

	*F*-statistic	*P* value	*df*	*η*^*2*^
Season	1.71	0.191	1	< 0.05
Year	68.12	< 0.001	3	< 0.05
Season:year	34.94	< 0.001	3	< 0.05

## Discussion

We used a combination of passive and active PIT tag antennas, along with the ability to tag and release large numbers of hatchery-reared fish, to robustly quantify movement of adult RGSM in a highly fragmented river. Quantifying movement patterns of small-bodied fishes in medium to large rivers is logistically challenging for several reasons, including limitations on the ability to tag small fishes, acquiring adequate re-encounter rates to describe movement patterns, the dynamic nature of lotic ecosystems, and short life-span of many small fishes. As our approach resulted in a relatively high re-detection rate and alleviates many of the shortcomings associated with traditional monitoring methods (i.e., does not require physical recapture), it could prove effective for other studies in large, open systems. Additionally, the data presented build upon emerging narratives regarding the movement ecology of PBS fishes and advance our ability to test movement theories in poorly understood species.

When interpreting our results, it is important to acknowledge limitations of the study. First, the behavior of hatchery-reared fish might not fully reflect that of wild RGSM; however, hatchery-reared fish are first generation offspring of wild-caught brood-stock. After initial stocking mortality, hatchery-reared RGSM survival is similar to wild fish [32]. Further, hatchery-reared fish are often found in the same isolated pools as wild fish [58], suggesting they exhibit similar behaviors. Second, our detection efforts were limited to tracking the movements of adult fish > 45 mm SL, and juvenile fish may be more likely to disperse upstream than adults, as documented in Pecos Bluntnose Shiner (*Notropis simus pecosensis*) in the Pecos River [25]. Future research including a broader range of age- and size-classes may elucidate movement patterns undocumented in our study. Third, the number of re-detections of individual fish limited our ability to estimate more complex metrics (i.e., home range size). Finally, this study took place over a broad spatial and temporal extent with large numbers of tagged fish in the last remaining habitat of RGSM, but also occurred in a highly modified riverscape, in terms of both fragmentation, flow, and habitat alteration, and so, might not fully represent movement ecology of this species under pristine conditions.

Neither of the two prevailing movement theories assessed in this study adequately described the patterns observed in RGSM. Rio Grande silvery minnow and other PBS minnows move substantially more than predicted by the RMP [18]. The model developed by Radinger and Wolter [15] has been used in other studies to predict expected dispersal of stream fishes (e.g., [59, 60]), and efforts to incorporate data from recent fish movement studies from more species into the ‘fishmove’ package model are warranted to ensure dispersal capabilities are not underestimated, particularly for smaller bodied fishes. Underestimation of dispersal could have negative consequences for conservation and restoration efforts by discounting the importance of connectivity among populations (e.g., [61]). Movements by RGSM were not as expected under the CCH, with biased upstream dispersal, as movements were mostly in a downstream direction, although 26% of movements were upstream. The CCH suggests organisms that experience downstream drift must compensate with upstream dispersal to maintain upstream populations [62]. Early work with aquatic macroinvertebrates suggested movements should be biased in an upstream direction to account for downstream drift of propagules [19], but later work built on simulations suggested any upstream dispersal component may be sufficient for population persistence [63, 64]. Observations of downstream movement by hatchery-reared RGSM following release are common [42, 44, 65], as well as by other hatchery-reared species [66, 67]. When we excluded detections within the first week of release to account for this immediate downstream tendency, movement patterns were largely unchanged, and movements were still consistently more common in the downstream direction.

While neither the RMP nor the CCH adequately described RGSM movement patterns, the metrics quantified in this study provide insight into the movement ecology of RGSM and may help define the appropriate scale of conservation efforts for other imperiled PBS fishes. Our results demonstrate the ability and predilection of some RGSM to make long-distance movements, matching results documented in other members of this reproductive guild [18, 26, 68]. For example, a mark-recapture study of Flathead Chub (*Platygobio gracilis*), a species with non-adhesive eggs [62], documented upstream movements up to 33 rkm, with farther upstream movement blocked by a dam [68]. Previous studies reported movement of considerable distances upstream by a few individual RGSM (25.2 rkm; [44]), and our results substantiate these observations. We documented RGSM moving farther upstream than previously recorded (48.0 rkm), and in contrast, this pattern was not limited to a few individuals. Additionally, RGSM demonstrated a leptokurtic movement distribution, with most individuals remaining near their initial release site (< 10.0 rkm), and a smaller portion of individuals dispersing farther away (> 100.0 rkm). This heterogeneous movement distribution is well documented in many other fish species [15]. Additionally, the upstream- or downstream-most detection point used to calculate detected range sizes was frequently located within ± 5 rkm of San Acacia Diversion Dam. This pattern indicates the presence of barriers likely reduces the extent of movement potential for many individual RGSM. We were surprised to document 198 unique upstream passages through the slightly open gates at the San Acacia Diversion Dam, with most upstream passages (*n* = 129) occurring during irrigation season, when instream flows are reduced. Prior to our study, upstream passage through San Acacia Diversion Dam by RGSM had not been reported. Although these upstream dam passages are impressive, they account for < 0.5% of the total number of individuals released in the study. Instream barriers artificially restrict movement for fishes and might even increase residency within populations over time (e.g., [69, 70]). Increasing connectivity by providing more efficient passage around barriers and ensuring adequate river flows during irrigation season would likely allow for fish to expand their movement potential and increase the number of individuals moving to repopulate upstream habitats.

Our results suggest RGSM movement patterns can be described as nomadic. Nomadic patterns of movement are common among animals in systems with high intra- and interannual environmental variability [70]. Nomadic patterns of movement emerge when resources are unpredictable in both time and space [71]. Although annual patterns of flow in the MRG are generally predictable, with high snowmelt driven spring runoff followed by low summer flows, daily or weekly flows in the contemporary MRG can be highly variable depending on winter snowpack, local summer precipitation, upstream storage, and agricultural demand. Flow conditions were variable throughout our study period, with high magnitude discharge in 2019 contrasted by extended low flows the following year (USGS 08354900). Mean daily discharge was positively related to the probability of movement, suggesting fish were more likely to move during periods of higher flow. Although we did observe a weak, negative relationship between mean daily discharge and the total distances moved by season, movement distances calculated from detections on days with the highest mean discharge (> 915 cms [cubic meters per second]) occurred in the lowest reaches of the study area. As such, it is likely these individuals were flushed downstream, outside of the detection area. Individual behavioral responses, rather than swimming ability, have been ascribed to the persistence of fishes under extreme variations in flow [72]. Further, it is unlikely that fishes would have perception of anything except their immediate surrounding environment or memory of previously visited areas, especially given the dynamic nature of a sand-bed river. Thus, movements may be uninformed, occurring at a relatively constant rate independent of patch conditions [73]. We documented very low rates of site fidelity among individual RGSM with > 10 unique detections (i.e., instances where individuals leave a location and return more than once), suggesting they were moving at a relatively constant rate over the course of one year. Nomadism driven by search behavior [70] in RGSM may result in individuals or shoals apparently moving erratically or randomly, yet habitat patches remain occupied because of high turnover within patches. Although this study lacks the temporal and spatial resolution to determine fine-scale movement dynamics, such movements would explain the large movements and low degree of site fidelity observed here, but wide distribution of both adults and young-of-year during summer drying [36].

The movement patterns observed in this study, along with a reproductive biology involving downstream transport of eggs and larvae, invokes the drift paradox for RGSM persistence. Resolution of the apparent drift paradox has important management consequences. There are several potential resolutions to the drift paradox in the absence of biased upstream dispersal of adults that could maintain persistence of upstream populations: (1) random diffusive dispersal of adults with enough upstream movement [74], (2) upstream dispersal of younger life-stages [25, 27], (3) habitat features retain drifting eggs and larvae within natal reaches (e.g., lower velocity mesohabitats), or (4) a combination of these. Indeed, Chase et al. [25] determined both retention and upstream movement of juveniles played a role in upstream population persistence of Pecos Bluntnose Shiner. Historically, drift distances of eggs and larvae were potentially much lower because of increased habitat complexity [33]. Under contemporary, degraded conditions, appropriate nursery habitats are nearly absent except at high flows, resulting in loss of eggs downstream and high variance in reproductive success among years [39]. Recruitment is minimal during low-flow years [5, 38], likely leading to negligible density-dependent dispersal. Further, upstream dispersers are blocked by diversion dams, resulting in ecological ratchet mechanisms [75], where upstream populations are extirpated, and colonizers from downstream populations can no longer disperse.

Each of these resolutions carry distinct management actions. Removal of longitudinal barriers would likely improve persistence by allowing upstream dispersal, regardless of life stage. If dispersal of juvenile fish is the primary driver of upstream population persistence, maintaining surface flows post-spawning in addition to fish passage will be necessary. However, effective conservation of nomadic species hinges on understanding movement rules for departure from patches and maintenance of connectivity among patches [6, 76]. The seasonal variation we documented in RGSM movement patterns can guide the timing of future release efforts to maximize retention efficiency among river reaches. Conversely, if persistence is primarily maintained through retention of eggs and larvae near natal areas, conservation efforts would be more effective if focused on restoring floodplain connectivity combined with environmental flows to improve retention and increase the carrying capacity. Persistence of PBS fishes via upstream dispersal of adults or retention of eggs and larvae has spurred substantial debate [77, 78]; we find it unlikely one occurs to the exclusion of the other given significant collections of larvae in upstream reaches in high-flow years [79] and empirical evidence that both occur in the same species at the same point in time [25]. However, persistence under contemporary flow and channel conditions may now rely more on the extreme upstream dispersers because of increased displacement distances with habitat simplification and many species have experienced vast range reductions [4, 34, 37].

Historically, PBS minnows were widespread, persisted through extreme environmental variation, and were often subjected to extended periods of intense drought [1, 47]. Their historical persistence likely required access to refugia during periods of drying and subsequent connectivity to allow recolonization of extirpated reaches. In contemporary riverscapes, many PBS fishes have suffered expansive range reductions because of the ratcheting effect of fragmentation and stream drying [4, 75, 80]. The nomadic movement patterns and ability to move long-distances documented in our study, combined with high reproductive effort and relatively short generation times, suggest RGSM would be able to recolonize habitats over relatively short temporal scales, if such movements were not blocked by dams or desiccated river reaches.

## Conclusions

Incorporating knowledge of movement patterns in context with unique life history strategies into the management of imperiled species is crucial for their recovery in highly modified and degraded river systems. Occupying 5% of its historical range, knowledge that RGSM make long distance movements upstream, a movement pattern blocked by diversion dams in many cases, highlights the importance of connectivity for the persistence of PBS fishes [1, 4, 75]. Given the widespread fragmentation of rivers, this research could serve as an important model that can be applied to imperiled small-bodied fishes in other fragmented systems. A greater understanding of how fishes are using fragmented rivers and their abilities to recolonize habitats can help guide future recovery efforts of riverscapes to ultimately achieve self-sustaining populations of native fishes.

### Supplementary Information

Supplementary Material 1.

## Data Availability

The dataset used and/or analyzed during the current study are available from the corresponding author upon reasonable request.

## Abbreviations

RMP: Restricted movement paradigm
PBS: Pelagic broadcast spawning
CCH: Colonization Cycle Hypothesis
RGSM: Rio Grande silvery minnow
MRG: Middle Rio Grande
PIT: Passive Integrated Transponder
PITPASS: Passive Integrated Transponder Portable Antenna Systems
km: Kilometer
mm: Millimeter
cm: Centimeter
rkm: River kilometer
SL: Standard length
TL: Total length
ANOVA: Analysis of variance
cms: Cubic meters per second
