# Supplementary material for: Movement patterns of a small-bodied minnow suggest nomadism in a fragmented, desert river
Source: Mov Ecol. 2024 Jul 31;12:52. doi: 10.1186/s40462-024-00490-w (PMC11293174; doi:10.1186/s40462-024-00490-w)
Supplement: Supplementary file 1 — Supplementary Material 1. [file 40462_2024_490_MOESM1_ESM.docx]

**Supplementary Material**

**Table S-1.** Rio Grande silvery minnow released between 2019 and 2022 in the Middle Rio Grande of New Mexico, USA showing the approximate coordinates of release locations by river kilometer (rkm).

| River kilometer (rkm) | Latitude | Longitude |
| --- | --- | --- |
| 139.4 | 33.87398 | -106.84942 |
| 166.2 | 34.09825 | -106.87983 |
| 180.9 | 34.21963 | -106.89277 |
| 182.9 | 34.23517 | -106.89954 |
| 183.0 | 34.23626 | -106.89852 |
| 183.1 | 34.23712 | -106.89716 |
| 186.0 | 34.25586 | -106.88885 |
| 191.5 | 34.27564 | -106.85446 |
| 211.1 | 34.4153 | -106.80054 |
| 227.4 | 34.54405 | -106.76368 |
| 248.6 | 34.71344 | -106.74721 |
| 250.9 | 34.73169 | -106.73983 |

**Table S-2.** Float trips conducted in the Middle Rio Grande of New Mexico, USA by year, showing the dates, extent and distance (rkm) of each float trip. Active float trips spanned 170.5 rkm of the Middle Rio Grande.

| Year | Date | Extent | Distance (rkm) |
| --- | --- | --- | --- |
| 2019 | Mar 20–31 | 123.9–192.6 | 68.7 |
|  | Jul 16–22 | 153.2–183.8 | 30.6 |
|  | Nov 20–25 | 154.0–191.5 | 37.5 |
|  | Dec 3–7 | 134.4–193.1 | 58.7 |
| 2020 | Dec 8–11 | 168.0–186.0 | 18.0 |
| 2021 | Mar 25–27 | 169.6–257.7 | 88.1 |
|  | May 16–23 | 169.3–257.7 | 88.4 |
|  | Jul 16–17 | 180.7–186.1 | 5.4 |
|  | Dec 8–14 | 170.4–294.4 | 124.0 |
| 2022 | Mar 14–17 | 180.9–294.4 | 113.5 |
|  | May 9–13 | 180.9–294.4 | 113.5 |

**Table S-3.** Results of binomial logistic regression assessing differences in Rio Grande silvery minnow (*n* = 8,264) movement by season. Fish that were only detected once after release were removed from analysis. Model estimates include a random effect of individual tag ID.

| Predictors | Estimates | *SE* | $\boldsymbol{\beta}$ | *P*-value |
| --- | --- | --- | --- | --- |
| Intercept | 0.18 | 0.05 | 0.06 | <0.001 |
| Mean Discharge | 2.54 | 0.19 | 0.19 | <0.001 |
| Body Length (SL, mm) | 1.01 | 0.00 | 0.04 | 0.093 |
| Season [Off] | 1.18 | 0.13 | 0.13 | 0.127 |
| Released [Below] | 4.01 | 0.37 | 0.37 | <0.001 |
| Days at Large | 1.07 | 0.00 | 0.18 | <0.001 |
| Year [2020] | 1.81 | 0.21 | 0.21 | <0.001 |
| Year [2021] | 1.15 | 0.19 | 0.19 | 0.397 |
| Year [2022] | 1.99 | 0.35 | 0.35 | <0.001 |
| Season [Off] * Released [Below] | 0.51 | 0.06 | 0.06 | <0.001 |

**Table S-4.** Results of zero-truncated lognormal linear mixed effects model (*n* = 4,689) assessing differences in Rio Grande silvery minnow total distances moved by season. Model estimates are provided on the log-scale and include a random effect of individual tag ID.

| Predictors | Estimates | *SE* | $\boldsymbol{\beta}$ | *P*-value |
| --- | --- | --- | --- | --- |
| Intercept | 0.01 | 0.10 | -0.05 | 0.95 |
| Mean Discharge | -0.16 | 0.04 | -0.10 | <0.001 |
| Body Length (SL, mm) | 0.21 | 0.03 | 0.13 | <0.001 |
| Season [Off] | 0.05 | 0.10 | 0.03 | 0.63 |
| Released Below | -0.24 | 0.09 | -0.15 | 0.01 |
| Days at Large | 0.03 | 0.00 | 0.40 | <0.001 |
| Year [2020] | -0.17 | 0.08 | -0.10 | 0.05 |
| Year [2021] | 0.02 | 0.15 | 0.01 | 0.90 |
| Year [2022] | -0.17 | 0.16 | -0.11 | 0.28 |
| Season [Off] * Released [Below] | 1.12 | 0.11 | 0.68 | <0.001 |


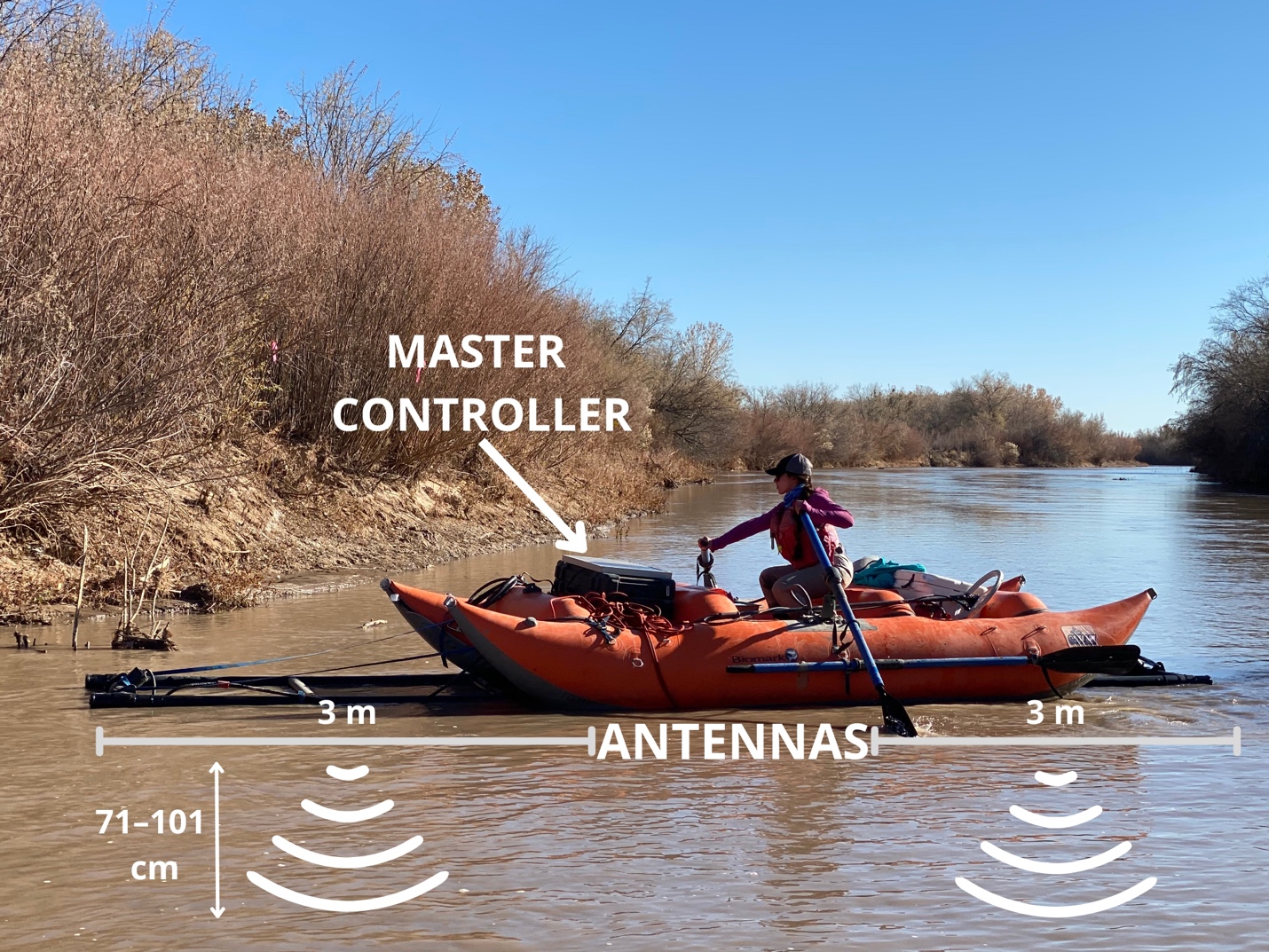


**Figure S-1.** Raft mounted Passive Integrated Transponder Portable Antenna Systems (PITPASS) in the Middle Rio Grande. The system consists of a raft to provide a platform for the floating antenna modules, a multiplexer to operate the antennas, an integrated GPS, and a data recorder. Two antennas are mounted under each raft measuring 1 m x 3 m each, totaling 6 m of coverage with read ranges of depths up to 101 cm.


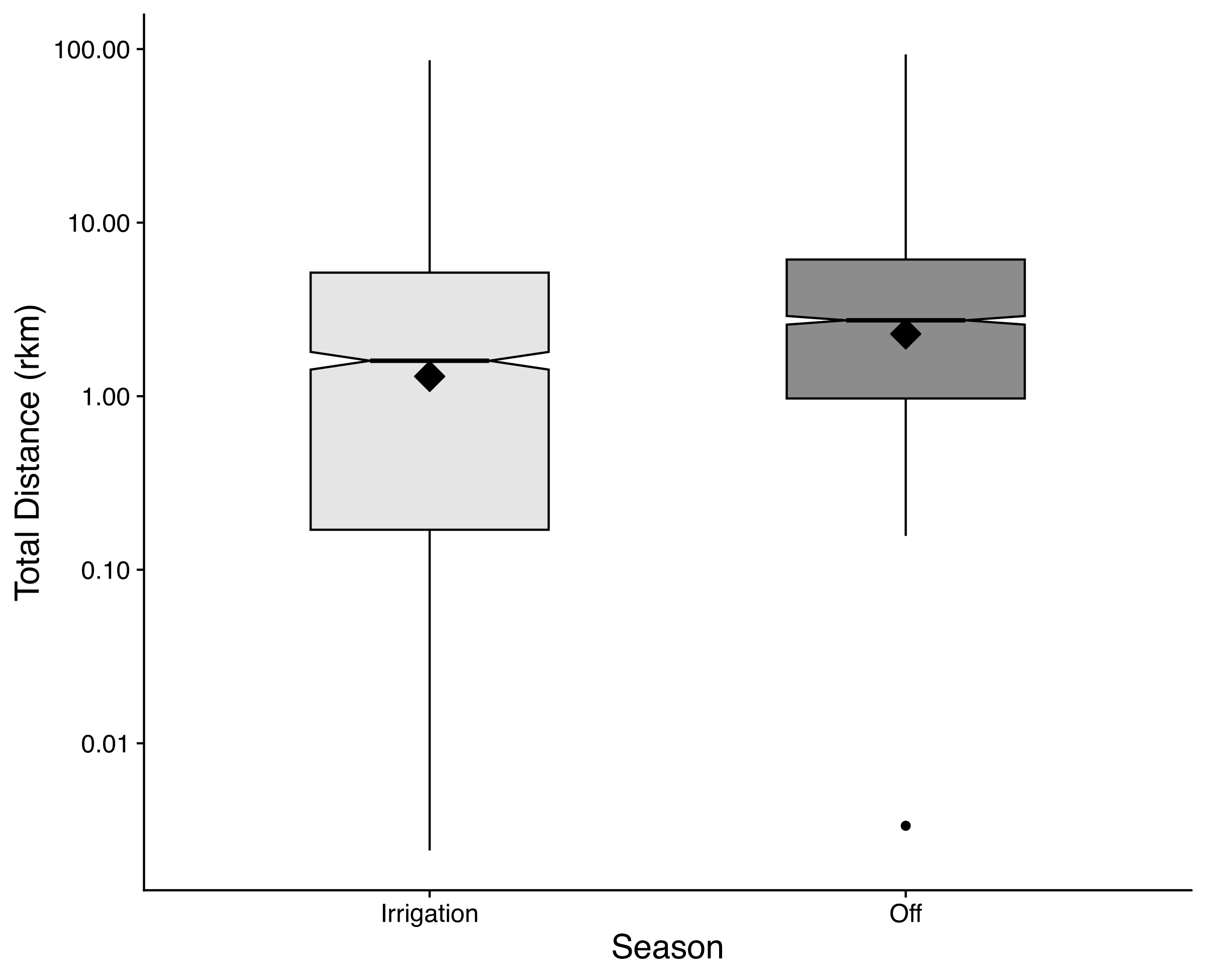


**Figure S-2.** Total distance moved (rkm, log_10_-scale) by PIT-tagged Rio Grande silvery minnow in the Middle Rio Grande by season (Irrigation = March–October; Off = November–February). Black diamonds represent the mean total distance moved (rkm) in each season. Boxes display the interquartile range with the bold line representing the median total distance moved (rkm), and whiskers extending to the largest and smallest value no further than 1.5x the interquartile range. The mean total distance moved was 1.5 rkm in the irrigation season and 1.8 rkm in the off season. The greatest maximum total distance moved by season occurred in the off season; however, the distributions of distances in both seasons greatly overlap.
